# Are IL-1 family cytokines important in management of sickle cell disease in Sub-Saharan Africa patients?

**DOI:** 10.3389/fimmu.2023.954054

**Published:** 2023-03-09

**Authors:** Liliane K. Siransy, Romuald S. Dasse, Honoré Adou, Patricia Kouacou, Sidonie Kouamenan, Yassongui Sekongo, Richard Yeboah, Charlene Memel, Aniella Assi-Sahoin, Salimata Y. Moussa, Doris Oura, Jocelyne Seri

**Affiliations:** ^1^ Immunology–Allergology Department, Medical Sciences, Felix Houphouet Boigny University, Abidjan, Côte d’Ivoire; ^2^ Transfusional therapeutic department, National Blood Transfusion Center, Abidjan, Côte d’Ivoire; ^3^ Immunology Department, CHU Bouake, Alassane Ouattara University, Bouake, Côte d’Ivoire

**Keywords:** sickle cell disease, cytokines, IL-1, IL-18, IL-33, Africa, chemokines

## Abstract

**Introduction:**

Sickle cell disease (SCD) is the most common genetic disease found in Africa and throughout the world. It is responsible for a high rate of hemolysis, systemic inflammation, and modulation of the immune system with the involvement of immunological molecules, such as cytokines. IL-1β is a major inflammatory cytokine. IL-18 and IL-33, members of IL-1 family, also exhibit characteristics of inflammation-related cytokines. Thus, in order to contribute to the evaluation of the severity and prognosis of SCD in Africa, this study aimed to estimate the cytokine response, in particular the levels of cytokines of the IL-1 family, in sickle cell patients living in a Sub-Saharan country.

**Methods:**

Ninety patients with a diagnosis of SCD were recruited with different hemoglobin types. Samples were assessed for cytokine levels using the Human Inflammation Panel assay from BioLegend. The assay allows the simultaneous quantification of 13 human inflammatory cytokines/chemokines, i.e., IL-1β, IFN-α2, IFN-γ, TNFα, MCP-1 (CCL2), IL-6, IL-8 (CXCL8), IL-10, IL-12p70, IL-17A, IL-18, IL-23, and IL-33.

**Results and discussion:**

the assessment of plasma cytokines in SCD patients revealed significantly increased levels of IL-1 family cytokines in crisis compared to steady state, suggesting a substantial involvement of these cytokines in clinical exacerbation. This suggests the possibility of a causal effect in the SCD pathology and can open the way to define better care, pointing toward new therapeutic avenues for sickle disease in Sub-Saharan Africa.

## 1 Introduction

Sickle cell disease (SCD) is the most common genetic disease in Africa, more than 23 countries in West and central Africa and throughout the world (1). Increasing global migration has introduced SCD into many areas where they were not originally endemic (1, 2), so that the disease has been designated by the World Health Organization (WHO) and United Nations as a global public health problem (1, 3). SCD is characterized by the formation of a particular hemoglobin called hemoglobin S (HbS), generated a single point mutation in the β globin chain of hemoglobin (Hb) that causes the substitution of the glutamate at position 6 with a valine (4). People who inherit the abnormal gene from both parents are homozygotes (SS or SSFA2) and develop SCD (5).

Other types of abnormal hemoglobin are frequently associated with HbS, e.g., hemoglobin C (HbC). Individuals with both HbS and HbC are heterozygotes HbSC (SC). Although the clinical complications of hemoglobin C disease are not severe, inheritance with other abnormal hemoglobin such as hemoglobin S may have significant consequences (6). Another frequent association described (SFA2*)* is the inheritance a thalassemia defect on one β gene that reduces the production of hemoglobin associated with the β S gene. Despite our understanding of the molecular basis and pathophysiology of these diseases, the burden is still heavy, particularly in countries that have medical settings with limited resources (4, 7).

The sickle cell gene is present in 10-45% of the population in many countries, resulting in an estimated prevalence of at least 2% (1). Clinical manifestations of SCD are of three main categories: chronic hemolytic anemia, vaso-occlusive phenomena and extreme susceptibility to infections that varies greatly from one individual to another. Deaths from SCD complications occur mostly in children under five, adolescents and pregnant women (1). Infant morbidity and mortality associated with SS homozygosity is very high among African children (50-90%) (8) and, under the age of five, it is related to acute, chronic and severe complications (9–11).

Inflammation is a fundamental component in SCD. HbS polymerization under deoxygenation conditions causes the red blood cells to adopt their characteristic sickle shape. This predisposes red cells to numerous intracellular and membrane alterations that make them more adhesive. Therefore, they interact with endothelial cells, activated neutrophils and platelets. Sickled red cells also release heme, which is highly inflammatory, and numerous other molecules with inflammatory potential, such as ATP, Cyclophilin A, and extracellular DNA (12). These molecules act as damage-associated molecular patterns (DAMPs) and participate in the activation of the innate immune system and the coagulation system. All these events participate in the chronic inflammatory processes in the SCD leading to numerous complications.

A very serious and dramatic complication is stroke, which causes severe motor and neurocognitive sequelae. The cerebrovascular accident exists in all forms of SCD (SS, SC, SFA2) ranging from 1.2 to 12% of prevalence according to the age of the patient and the country (13). By the age of 45, about 25% of patients have suffered an apparent stroke even with chronic transfusion therapy or a normal transcranial doppler (TCD) (13–15). In addition, after a first stroke, silent cerebral infarcts can occur despite regular blood transfusions (14). Although in SCD the incidence of first clinical stroke has fallen over the past decades in the USA and Europe, this is still a highly prevalent matter of concern in Africa, where the majority of people with this condition live. Prevention and care of stroke as well as other complications have been largely neglected in Africa. To improve quality of life, prevention and early recognition *via* cytokines may be a key to decrease morbidity and mortality in SCD. The involvement of immune reactions and their effector molecules, such as the cytokine response in SCD, are of increasing interest, due to cytokines’ capacity to drive acute and chronic inflammatory states, thereby contributing to the pathology of SCD (16–18). Several cytokines and chemokines are involved in the chronic inflammatory state in SCD patients. Increased IL-1α and IL-1β are reported in SCD, which trigger inflammatory reactions leading to primary leukocyte recruitment, endothelial cell activation and production of other many inflammatory mediators, in particular IL-8. IL-8 is a very important chemokine, which facilitates the recruitment of leukocytes, such as neutrophils and eosinophils, to blood vessel walls (19, 20). The increased production of inflammatory cytokines could contribute to the pathophysiology of the disease and can be responsible for the damage to the different organs and the occurrence of common cyclic events in sickle cell patients (17, 21). Insightful understanding of the correlation between inflammatory mediators, such as cytokines, and stroke or other organ damage may provide new biomarkers or therapeutic approaches for SCD management.

Cytokines of the IL-1 family have multiple local and systemic effects, which regulate both innate and adaptive immune responses. Many IL-1 family cytokines have inflammatory activity (IL-1α, IL-1β, IL-18, IL-36α, IL-36β, IL-36γ), while others are mainly anti-inflammatory (IL-1Ra, IL-33, IL-36Ra, IL-37, IL-38) (22). IL-1β is a major inflammatory cytokine that has been associated with exacerbation of injury in stroke, but that was shown to exert both deleterious and protective functions in stroke (19).

Most studies involving cytokines have been conducted in high income countries. Unfortunately, in West Africa, access to such analysis is not possible due to lack of well-equipped infrastructures, absence of standardized management and too few skilled immunologists.

To our knowledge, very few studies have investigated the cytokines association with SCD in our area. This study is the first one done in sickle cells patients in Côte d’Ivoire and will help the evaluation of the significance of cytokines, in particularly those belonging to the IL-1 family, in the management, severity and prognosis of SCD in Sub-Saharan Africa.

## 2 Material and methods

### 2.1 Study population

This is a prospective case-control study of a population of 90 patients with SCD, aged 4 to 55 years, and followed at the Therapeutic and Research Unit of the National Blood Transfusion Center in Abidjan, Côte d’Ivoire. It was conducted after approval by the national ethics committee (n°44_2018-SIHIOTS). Informed consents were obtained from the patients’ parents or legal guardians before patients were enrolled in this study.

Patients were divided into two groups: patients admitted for crisis for and steady state patients.

Steady state was defined as a state without pain, illness, or infection during the last three months before the study; this group represents our control group compared with the crisis group.

The profile of homozygous or heterozygous sickle cell patients was determined by hemoglobin electrophoresis on cellulose acetate strips (pH 9.2).

Patients with crisis were admitted to the Unit, while those who were asymptomatic, i.e., at steady stat,e were present to the Unit of Therapeutic Transfusion for routine monitoring. Crisis was defined as an episode of acute diffuse pain with infection or anemia, requiring hospitalization and/or analgesic administration. Clinical investigations were evaluated by a specialist in hematology. Cytokine levels were compared in steady-state and crisis patients according to the type of hemoglobin and the complications. All subjects were from West African countries: Côte d’Ivoire, Ghana, Guinea, Mali, Nigeria and Togo.

### 2.2 Cytokine analysis

Blood samples were collected by venipuncture in EDTA tubes for basic determination of hematological indices and for cytokine dosage.

The blood count was obtained with an automated hematological cell count machine (Sysmex XN 550). Plasma was extracted by centrifugation at 1000xg at 4°C for 10 min and stored at -30°C for cytokine assays.

Before assay, samples were thawed, mixed and centrifuged, then assessed for cytokine levels using the BioLegend LEGENDplex™ Human Inflammation Panel assay (Cat. N°740118), an immunoassay based on immunofluorescent beads. The assay allows the simultaneous quantification of 13 human inflammatory cytokines/chemokines, i.e., IL-1β, IFN-α2, IFN-γ, TNFα, MCP-1 (CCL2), IL-6, IL-8 (CXCL8), IL-10, IL-12p70, IL-17A, IL-18, IL-23, and IL-33. The assay was performed in the BioLegend laboratory in San Diego, CA, USA.

All samples were processed and analyzed on the same day of thawing. A minimum of cytokine-specific 3000 beads were acquired with a BD FACSCalibur™ cytometer. Controls provided by the manufacturer were used. Data analysis was performed using LEGENDplex™ Data Analysis Software.

### 2.3 Statistical analysis

All results are expressed as mean ± SD. Data were analyzed using the SPSS version 22.0 program (SPSS Inc., Chicago, IL, USA). Statistical results with a *p* value ≤ 0.05 were significant.

For the comparison of cytokine means, we first used the Levene test to determine the equality of variances: if the Levene test was not significant (*p* > 0.05), we had an equality of variances and used the Student *t*-test for the 2-modality variables and the ANOVA test for the more than 2-modality variables. If the Levene test was significant (*p* ¾ 0.05), we had a variance inequality and in this case we use nonparametric tests such as the Mann-Whitney U test for the 2-modality variables and the Kruskal-Wallis for variables with more than 2 modalities.

## 3 Results

### 3.1 Biometric and epidemiological characteristics of sickle cell patients by clinical status

Ninety (90) patients with a diagnosis of SCD, comprising 48 women (53.3%) and 42 men (42.70%) were recruited.

Among the sickle cell patients, 34 (37.8%) were in steady state phase and represented our stable controls, while 56 (62.2%) were in crisis and represented the cases.

Our SCD patients show in general a low body mass index BMI (**Table 1**). We find an average BMI of 20.74 kg/m^2^ ± 2.82 in SCD patients in crisis versus 20.24 kg/m^2^ ± 5.63 in SCD patients in steady state phase. Few patients were overweight, and none was obese.

**Table 1:** Demographic and hematological characteristics of sickle cell patients.

Parameter	Steady state	Crisis	*p v*alue
Age (years) *			
All patients	20.9 ± 14.6 (4–50)	27.3 ± 13.1 (4–55)	0.09
4-9	6.3 ± 2.1	6.6 ± 1.7	
10-19	17.5 ± 0.7	15.1 ± 3.6	
20-29	23.6 ± 3.4	23.5 ± 3.2	
30-39	33.5 ± 4.4	34.5 ± 3.1	
40-55	44.3 ± 4.3	48.2 ± 4.4	
Sex ratio (M/F)	30.7	1.0	
BMI (kg/m^2^) *	20.2 ± 5.6 (7.6-27.3)	20.7 ± 2.8 (13.7-26.2)	0.36
Hemoglobin (g/l) *	8.7 ± 2.0 (5.6-3.1)	8.5 ± 2.1 (4.8-14.3)	0.64
Hemoglobin type (%) **			
SSFA2(SS)	51	51.2	0.96
SFA2	29.4	32.8	0.68
SC	19.6	16	0.42
WBC (cells/μl) *	10431.3 ± 4631.4 (5100–18600)	10393.5 ± 4129.4 (3600-20600)	0.97
Lymphocytes (cells/μl) *	3691.2 ± 1305.7 (1769.7-5952)	4248.2 ± 1482.9 (1945.8-8049.6)	0.09
Neutrophils (cells/μl) *	5521.8 ± 3432.2 (623-11346)	5078.7 ± 1852.6 (1427.4-8793.4)	0.56
Monocytes (cells/μl) *	1225.1 ± 858 (316.2-4018)	1375.7 ± 2003.7 (285.2-10046.4)	0.69

*Mean ± SD (min-max).

**Mean percentage of hemoglobin types among all SCD patients (steady state and crisis) were: SSFA2, 51.1%; SFA2, 31.1%; SC, 17.8%.

**Figure 1:**
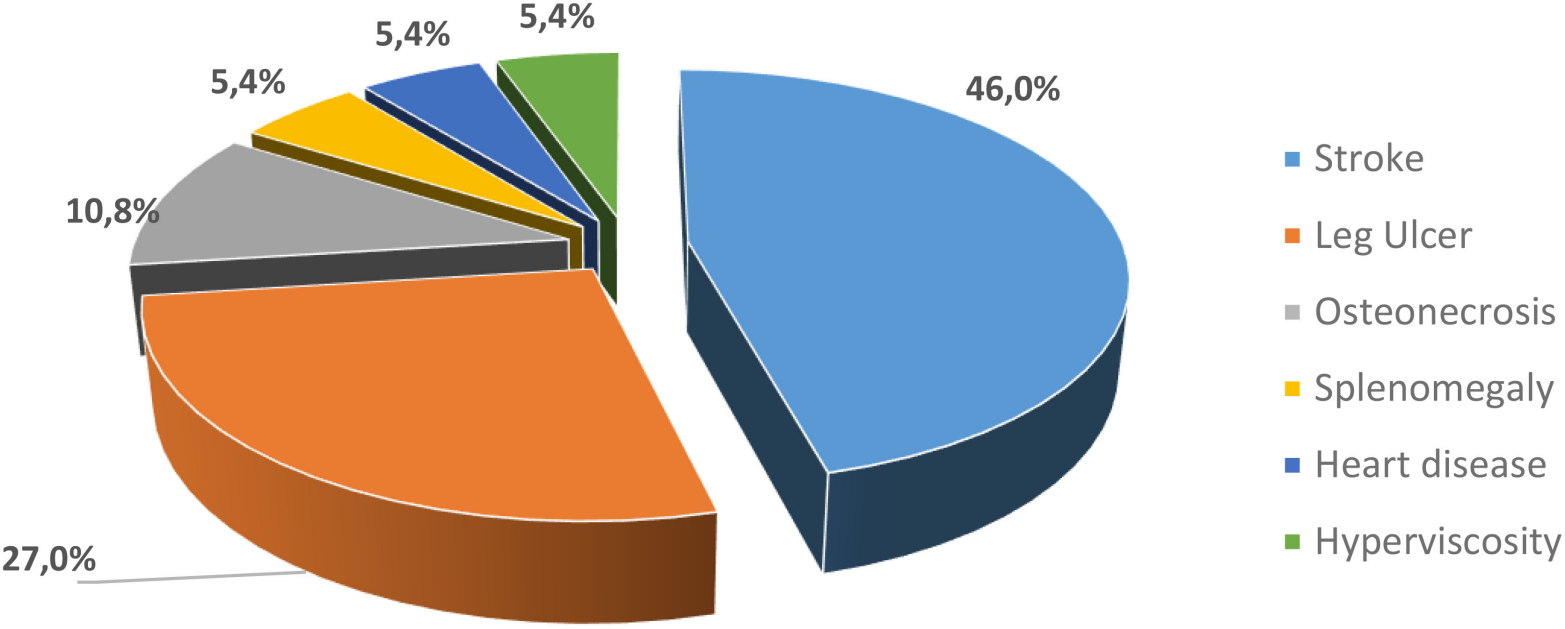
Frequency of complications in our SCD patients .

Cerebral stroke was found in 45.95% followed by leg ulcer (27.03%) and osteonecrosis (10.81%). Other complications such as heart disease, splenomegaly and hyperviscosity were found in patients in our study with prevalence of 5.41% (**Figure 1**). The minimum age for stroke patients was 7 years, with an average of 21 years and a maximum of 37 years.

### 3.2 Biological characteristics

#### 3.2.1 Type and levels of hemoglobin and white blood cells

Regarding hemoglobin type, 46 were homozygous Hb SS (SSFA2) (51.1%), 28 were heterozygous SFA2 (31.1%) and 16 were Hb SC (17.8%) (**Table 1**).

SCD was associated with a decrease in the hemoglobin level in the 2 groups, steady state and crisis (8.7 ± 2 and 8.5 ± 2.1 g/l, respectively), compared to the normal blood level of hemoglobin but showing a non-significant difference (*p* = 0.64) (**Table 1**).

No significant difference was observed for white blood cells and leukocyte subpopulations for SCD in steady state phase or during crisis.

#### 3.2.2 Cytokines in steady state and crisis

Thirteen cytokines were measured in our SCD patients. Three cytokines of the IL-1 family were assessed, i.e., IL-1β, IL-18 and IL-33. We have compared their levels to those of the most studied cytokines in SCD, i.e., TNFα, IL-6, IL-8 and IL-10. They were all increased in the crisis group except IL-10 and IL-33, with TNFα and IL-10 not reaching statistical significance (**Table 2**). By contrast, anti-inflammatory IL-33 and IL-10 were decreased in the crisis group, with significant difference for IL-33 (**Table 2**). Other cytokines (IFN-α2, IFN-γ, MCP-1, IL-12p70 and IL-17A, IL-23) were detected, which however did not differ between groups (**Supplementary Table 1**).

**Table 2:** General cytokines profile of the SCD patients.

Cytokines *(pg/ml)*	Steady state *mean ± SD (min-max)*	Crisis *mean ± SD (min-max)*	*p* value
IL-1β	3.50 ± 6.65 (0.00-27.26)	43.21 ± 181.42 (0.00-974.33)	0.03
IL-18	667.82 ± 1049.31 (48.76-2879.67)	1433.02 ± 1901.54 (96.18-9130.03)	0.01
IL-33	13.42 ± 29.18 (0.00-93.23)	6.13 ± 13.60 (0.00-28.45)	0.04
IL-6	53.26 ± 121.18 (0.00-473.4)	506.17 ± 2461.94 (0.00-13059.41)	0.01
IL-8	294.17 ± 671.05 (0.00-10693.45)	1133.98 ± 2602.06 (0.00-12000)	0.0024
IL-10	14.23 ± 25.73 (0.00-96.04)	8.78 ± 12.20 (0.00-57.85)	0.1
TNFα	2.03 ± 2.81 (0.00-15.08)	3.36 ± 9.80 (0.00-49.91)	0.2

We also compared the mean levels of circulating cytokines in patients during crisis with those in steady state relative to the type of hemoglobin (**Table 3**).

**Table 3:** Comparison of circulating cytokines for HbSSFA2, HbSFA2 and HbSC steady state and crisis patients.

Cytokine *(pg/ml*)	Hb Type	Steady state *mean ± SD (min-max)*	Crisis *mean ± SD (min-max)*	*P value*
	SSFA2	9.3 ± 16.7 (0-27.3)	6.74 ± 13.1 (0-46.1)	0.28
IL-1β	SFA2	2.0 ± 1.4 (0-3.7)	60.4 ± 221.8 (0-974.3)	0.02
	SC	3.6 ± 4.2 (0-10.8)	1.9 ± 2.7 (0-6.6)	0.21
	SSFA2	868.6 ± 1388.8 (96.2-2879.7)	1394.7 ± 1337.5 (95.3-4091.3)	0.16
IL-18	SFA2	728.1 ± 749.7 (48.8-1759.2)	1388.3 ± 2069.8 (167.3-6806)	0.19
	SC	298.3 ± 256.8 (106.1-774.8)	1941.6 ± 4018.5 (120.1-9130)	0.01
	SSFA2	26.2 ± 38.2 (0-93.2)	3.0 ± 9.0 (0-29.4)	0.000096
IL-33	SFA2	4.6 ± 8.5 (0-18.4)	3.6 ± 9.3 (0-28.5)	0.33
	SC	34.7 ± 19.6 (0-46.2)	0.0 ± 0.0	0.000029
	SSFA2	2.0 ± 4.2 (0-15.8)	2.7 ± 3.3 (0-9.3)	0.28
TNFα	SFA2	0 ± 0	2.6 ± 11.5 (0-49.9)	0.23
	SC	4.8 ± 4.7 (0-10)	2.6 ± 2.3 (0-4.7)	0.11
	SSFA2	69.6 ± 158.1 (0-473.4)	41.8 ± 82.4 (0-309.1)	0.21
IL-6	SFA2	22.0 ± 46.5 (0-28.7)	2618.1 ± 55.0 (0-13059.4)	0.000035
	SC	14.1 ± 16.2 (0-28.1)	55.1 ± 84.8 (0-230.7)	0.12
				
	SSFA2	640.9 ± 1110.6 (3.5-3499.8)	1662.1 ± 3630.1 (0-12000)	0.0042
IL-8	SFA2	492.2± 1126.1 (0-10693)	2413.8 ± 5052.4 (0-344.5)	0.15
	SC	172.3 ± 198.9 (0-344.5)	345.2 ± 438.2 (3.3-1124.5)	0.12
	SSFA2	12.2 ± 17.7 (0-56.5)	8.8 ± 14.1 (1.8-57.9)	0.23
IL-10	SFA2	10.6 ± 14.5 (0-36.9)	9.3 ± 11.1 (0-29.8)	0.43
	SC	19.1 ± 36.1 (0-96.0)	5.1 ± 5.9 (0-11.9)	0.22

In SSFA2 patients, a significantly a lower level was measured for IL-33 (*p*=0.000096) during crisis. In SFA2 patients, a higher level was observed for IL-1β (*p*=0.02) during crisis. In SC patients, higher level of IL-18 (*p*=0.01) and lower values for IL-33 (*p*=0.000029) were observed during crisis.

#### 3.2.3 Cytokines in SCD complications

Complications have been observed in our SCD patients. Stroke was the most important (46%), followed by leg ulcer (27%) and osteonecrosis (10.8%) (**Figure 1**).

**Figure 2:**
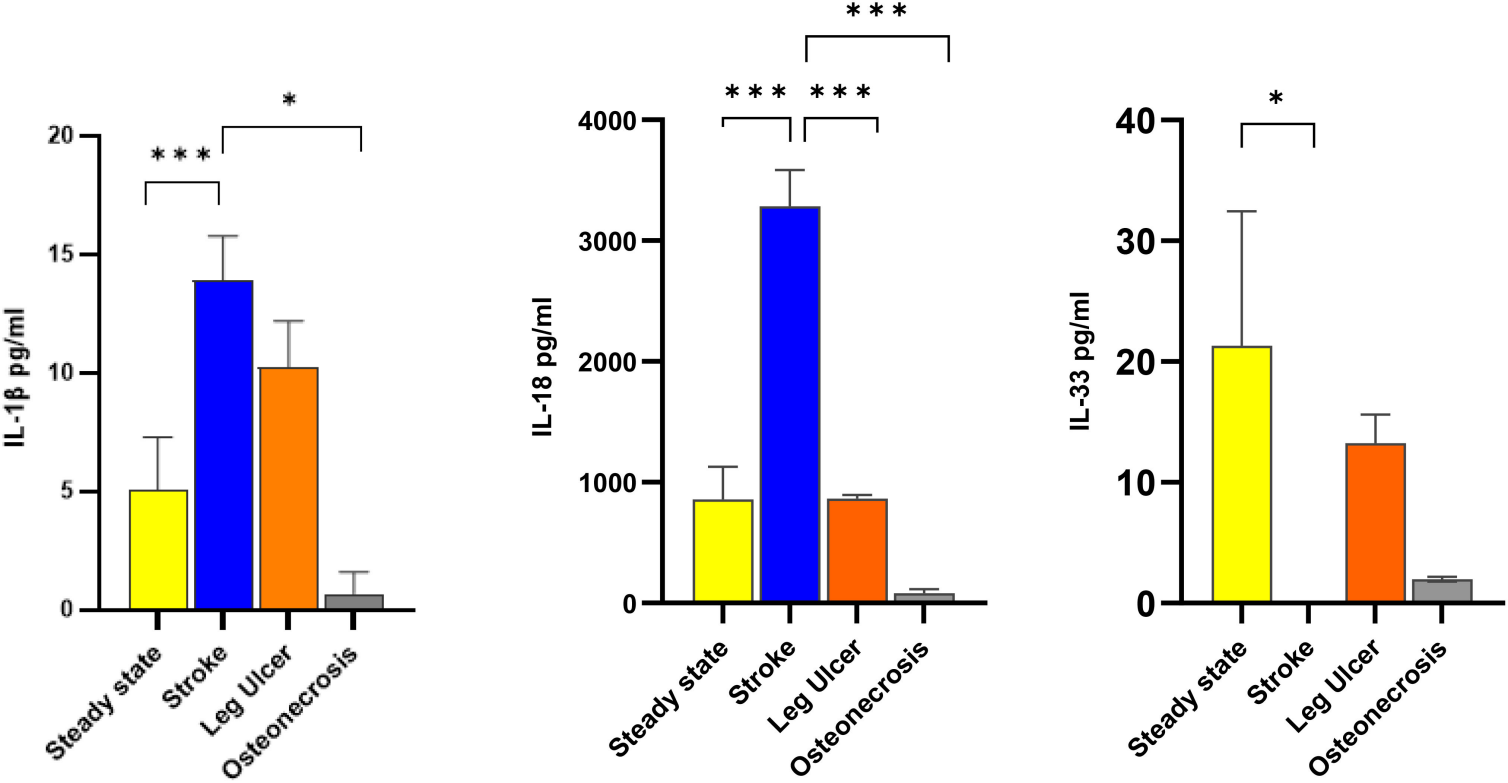
Comparison of IL-1 cytokine levels according to the 3 most common complications Cytokines levels were assessed in steady state SCD patients (34 patients; yellow columns), in crisis patients with stroke (17 patients; blue columns), in leg ulcers (10 patients; orange columns) and in osteonecrosis (4 patients; grey columns). Data are presented as mean pg/ml ± SD of IL-1β (left panel), IL-18 (center panel) and IL-33 (right panel). Statistical significances for complications vs. steady state were found for IL-1β (stroke vs. steady state, stroke vs. osteonecrosis), IL-18 (steady state vs. stroke, stroke vs. leg ulcer, stroke vs. osteonecrosis) and IL-33 (steady state vs stroke.). * p<0.05; *** p<0.001.

IL 1β and IL-18 were increased in stroke (*p*<0.001 stroke vs. steady state), while IL-33 was decreased (*p*<0.05 stroke vs. steady state) (**Figure 2**). In patients with osteonecrosis there was a tendency towards decreased levels of all three cytokines compared to steady state, which however did not reach statistical significance (**Figure 2**). In patients with leg ulcers the three cytokine levels did not significantly differ from those in steady state, although there was a tendency towards decrease (**Figure 2**). For the other cytokines, no significant difference was found (**Supplementary Figure 1**).

We also compared cytokines by type of crisis (hemolytic anemia, HA; vaso-occlusive crisis, VOC).

**Figure 3:**
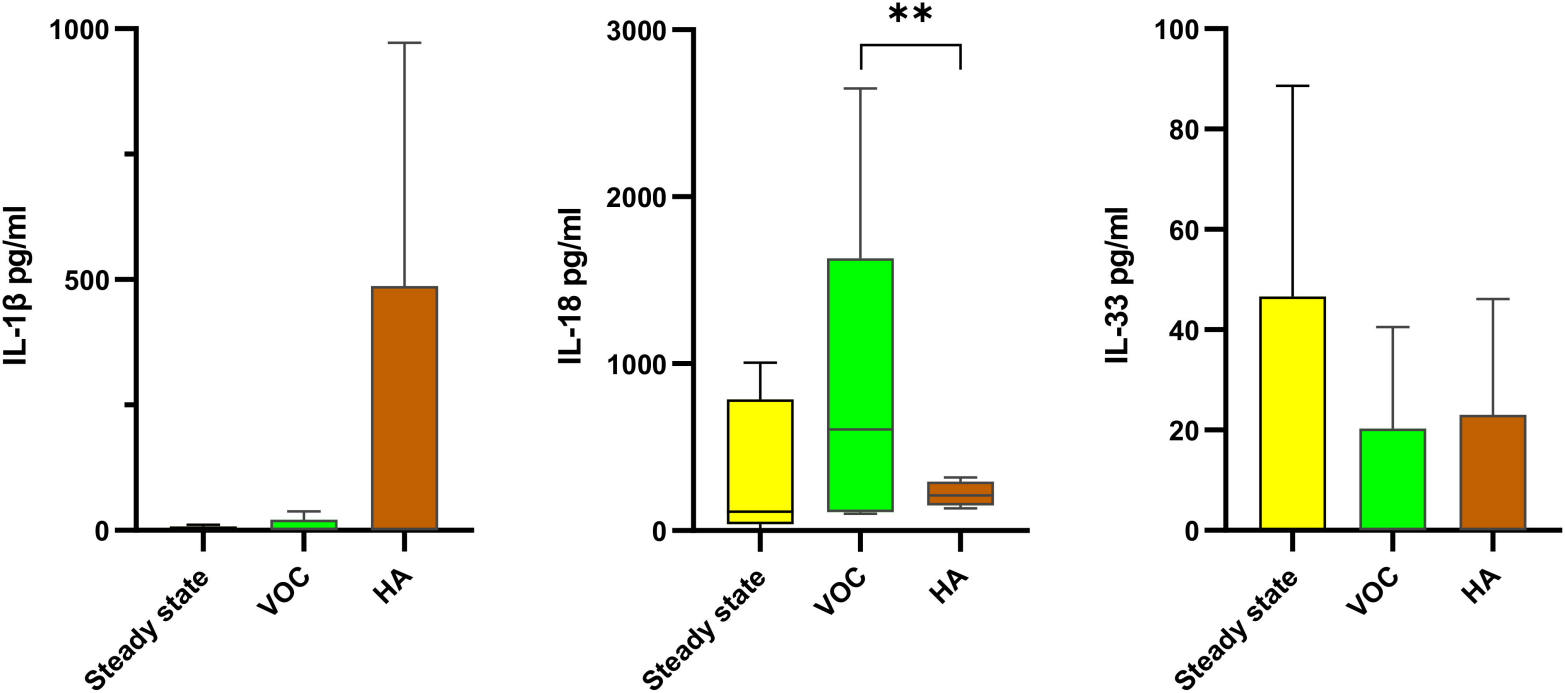
Comparison of IL-1 family cytokines according to the type of crisis in SCD patients The levels of IL-1β (left), IL-18 (center) and IL-33 (right) in SCD patients were compared between steady state (34 patients; yellow boxes), vaso-occlusive crisis (VOC; 24 patients; green boxes) and hemolytic anemia (HA; 32patients; brown boxes). Data are presented as median values and interquartile range in pg/ml. Statistical analysis was performed using Kruskal-Wallis. ** p<0.01 VOC, vaso-occlusive crisis; HA, hemolytic anemia.

Among the crisis, HA accounted for 55%, followed by VOC (45%). A tendency (not statistically significant) towards higher levels of IL-1β, IL-6, IL-8, and TNFα and lower levels of IL-10 were observed in HA, compared to steady state, while IL-18 tended to be higher in VOC (**Figure 3**).

## Discussion

In the present study, assessment of plasma cytokines in SCD patients revealed measurable circulating levels of the IL-1 family cytokines in both clinical presentations, crisis, and steady state (**Table 2**). We consider the cytokine levels in plasma of SCD patients in steady state as a benchmark, in order to assess the variations during crisis or with complications. How much these values compare to cytokine levels of healthy subjects is currently unknown (study ongoing), although from previously published data steady state SCD patients seem to have increased levels of IL-6, IL-8 and IL-18 (which in normal subjects are ≤ 10 pg/ml for IL-6 and IL-8, and around 200 pg/ml for IL-18), suggesting a partially upregulated inflammatory level in steady state SCD. Variability of conditions in different geographical settings and differences in detection assays makes impossible any quantitative comparison with data from other studies (23).

Patients in crisis showed higher levels of the inflammatory cytokines IL-1β, IL-6, IL-8 and IL-18 compared to those in steady state conditions and decreased levels of the anti-inflammatory factor IL-33 (**Table 2**). Differences were not statistically significant for IL-10 and TNFα.

IL-1β is a key mediator of the inflammatory response and shows a huge complexity. In addition to being essential for host response and resistance to pathogens, it also exacerbates the damage caused by chronic disease and acute tissue injury, as is the case in SCD (16, 24). Our study shows a significant increase of IL-1β levels in crisis subjects (*p*=0.03). The values found in sickle cell patients in crisis were 10 times greater compared with the steady state, suggesting an involvement of this cytokine in SCD crisis. Our values are similar to those observed by Pitanga et al., who found low IL-1β levels (below 10 pg/ml) both in normal children and in steady state SCD patients (18). The substantial increase in crisis patients that we have observed is in contrast to the data of Pathare et al., who found no change between steady state and crisis levels (24). It should be noted that the circulating IL-1β levels in healthy subjects reported in that study are exceedingly high and different from the low-undetectable levels commonly observed in healthy people (18, 25, 26), thus posing some questions on the reliability of the detection assay.

Although IL-1β has been well characterized, IL-18 and other members of the IL-1 family are less so (27, 28). In our study, IL-18 was detected at high levels both in steady state and, at even higher levels, during crisis. A study suggested a possible role for IL-18 in the physiopathology of crisis in SCD (29). High levels of IL-18 have been associated with high levels of LDH and uric acid, and patients with these indicators have poor prognosis (29). It should be noted that IL-18 is not an exclusively inflammatory cytokine (28). Its circulating levels are well detectable in healthy subjects and increase in essentially all inflammatory conditions (25, 28). IL-18 is an established biomarker of heart failure, so that it has been proposed that anti-IL-18 treatment may have therapeutic effects in patients with SCD at risk for cardiac arrhythmias and adverse outcomes (30).

Interleukin-33 (IL-33), another member of the IL-1 cytokine family, is widely expressed by various tissues, such as smooth muscle cells, epithelial cells, fibroblasts, and keratinocytes. IL-33 is mainly involved in type 2 inflammation, alternative to classical type 1 inflammation, and has anti-inflammatory protective effects in many diseases (31–33). In our study, IL-33 was significantly higher in steady state (**Table 2**). IL-33 has been proposed as a novel contributor to anemia in inflammatory disease through its effects on differentiation of erythroid cells and as a therapeutic target for this type of patients (34). This cytokine has been rarely investigated in SCD. A link between hemolysis and IL-33 has been proposed, although results might be biased by the fact that the evaluation was performed on stored red blood cells (35). In our study, IL-33 seems to behave as an anti-inflammatory cytokine. This may be due to the heterogeneity of patient genotypes, treatment, environmental impact, socio-economic status, nutrition, the presence of other associated inflammatory factors such as infections or other inflammatory stress and genetic polymorphism (36, 37).

SCD is characterized by common acute and chronic complications. In our study, five types of complications have been identified: stroke (46%), leg ulcer (27%), osteonecrosis (10.8%), hyperviscosity (5.4%), heart disease (5.4%), and splenomegaly (5.4%) and no patient has no more than one complication.

Stroke was the most important complication in our study and in other regions of the world (13) as it is a devastating complication and an important cause of death in SCD. Between 5 and 17% of SCD patients will suffer a first stroke during childhood or adolescence in the absence of screening and prophylactic treatment (10, 11, 13). The minimum age of stroke of our patients in our study was 7 years, a quite young age, and this could be explained by the precariousness of the management, lack of access to care, and inadequate plans for the management of sickle cell children (1). IL-1β has been associated with exacerbation of injury in stroke and has been implicated in the pathogenic processes associated with a number of central nervous system disorders (38–40), while IL-6 and IL-10 have been found to be neuroprotective (38). The mechanism by which IL-1β affects seizure is unknown. It is known that IL-1β administered with the excitatory neurotransmitter AMPA (α-amino-3-hydroxy-5-methyl- 4-isoxazole propionic acid) produces a widespread cortical cell death (38). Also, recent work in a murine model with SCD has shown that Interleukin-1 receptor inhibition reduced the size of stroke (41). Although there are indications of a detrimental role of IL-1β, a study showed that an increased IL-1β concentration was linked with protection from stroke development in HbSS children with abnormal transcranial doppler (TCD) (19). IL-1β has been therefore qualified as both beneficial and deleterious in cerebral ischemia depending on its plasma levels. Protective concentration ranges should be well identified to benefit from this predictive biomarker in the management of cerebrovascular events in SCD (19). This suggests that plasma IL-1β levels in combination with TCD measurements may be used to improve evaluation of stroke risk in HbSS patients, by early identification of those needing intensive prophylactic interventions, especially as these strokes can be asymptomatic or silent.

Sickle cell leg ulcers came in second position in our study (27.03%). It is the most important common cutaneous complication in SCD, severe, chronic, and disabling with intense and continuing pain (42). There is no official recommendations for treatment and a high rate of relapse (42). No significant differences was found for IL-1β or for any other cytokine, suggesting that these cytokines are not useful predictors for poor outcomes (**Supplementary Figure 1**) (43).

Osteonecrosis was the third most frequent complications in our study, with a prevalence of 10.81%. It is a form of ischemic bone injury that leads to degenerative joint disease, and SCD is an important cause of its occurrence (44). An estimated 50% of adults will develop an osteonecrosis by age 35 years old (45). The circulating IL-33 concentration is considered a biomarker of avascular necrosis of the femoral head in patients without SCD, since increased IL-33 levels correlate with osteonecrosis of the femoral head (46, 47). In our study, the IL-33 values in patients with osteonecrosis were very low, thus discounting this marker as an element of early diagnosis and progression of osteonecrosis. Our findings are consistent with those of Agrawal et al. in India (46), in which IL-33 plasma levels did not correlate with osteonecrosis of the femoral head in patients with SCD.

We also presented the results according to the hemoglobin type and the clinical status at the time of the blood sampling, to find a correlation between the hemoglobin type, the level of cytokines and the crisis (**Table 3**). Relative to the cytokines of the IL-1 family, significant differences between crisis and non-crisis states were found for phenotypes SFA2 (IL-1β), SSFA2 (IL-33) and SC (IL-18, IL-33). It is however difficult to determine an influence or a cause-effect correlation.

The clinical manifestations of SCD are multifaceted but we investigated the major features: vaso-occlusive (VOC) and hemolytic anemia (HA). In our study, HA was the most common with 55% followed by the VOC (45%). The lower frequency of VOC compared to other studies (48, 49) may be explained by the fact that VOC are mostly managed at home by patients and often not reported (50). We noted a tendency towards higher levels of IL-1β, IL-6, IL-8 and TNFα in HA compared to VOC, which however did not reach statistical significance, while IL-18 and IL-10 are significantly higher in VOC. None of them was however statistically different from the steady state levels. Since hemolysis triggers inflammation and participates in numerous multisystemic complications of SCD (51), and red blood cells could play a role in cytokine signaling (52), this could explain why inflammation-related cytokines are higher in HA. Conversely, the anti-inflammatory cytokine IL-10 was significantly increased in VOC compared to HA, in agreement with other studies (53, 54). The fact that IL-18 was also higher in VOC underlines the notion that this is not a classically inflammatory cytokine and supports previous indirect data hypothesizing the involvement of IL-18 in vascular occlusion in SCD (28). Furthermore, data in SCD mice showed that IL-18 is strongly involved in the entire process of leucocyte recruitment in VOC, while IL-1β is only involved in the late steps of the process (55).

Therapeutic options for SCD are limited to hematopoietic stem cell transplantation, which is a curative option but not available for our countries due to its high cost and the lack of resources. Therefore, anti-inflammatory drugs are important for us because they can neutralize key players in the SCD inflammatory scenario. Many are currently under investigation as potential therapeutics, and agents such as antibodies to anti-IL-1β, the IL-1 receptor antagonist or the IL-18 binding protein are expected to provide benefits for these patients (12).

In conclusion, few studies in Sub-Saharan countries describe the cytokine levels in SCD patients. Our data emphasize the role of inflammation in SCD in crisis, suggesting an important involvement of inflammation-related cytokines in the dynamics of SCD.

IL-1 family members are poorly studied in SCD, even though these cytokines are key mediators of the inflammatory response that enhances the damage caused by SCD. IL-1 family cytokines deserve further consideration with larger cohorts, as they can be useful predictors of poor or favourable outcome, depending on the circumstances. A better knowledge of these cytokines in the evolution of SCD in African countries, considering the specific aspects of patients’ management due to extrinsic factors (such as the environment, access to care, socio-economic status), will improve survival and increase the quality of life of these patients in low-income countries.

## Data availability statement

The original contributions presented in the study are included in the article/**Supplementary Material**. Further inquiries can be directed to the corresponding author.

## Ethics statement

The studies involving human participants were reviewed and approved by Ethic Commitee of Faculty of Medecine-SIHIOTS 044_2018-sihiots. Written informed consent to participate in this study was provided by the participants’ legal guardian/next of kin.

## Author contributions

LS conceived and designed the experiments, conducted all the experiments, analyzed, and interpreted the data, assembled all the figures, supervised the study, and wrote the manuscript. RD co-supervised the study, contributed to the scientific discussion, and wrote the manuscript. HA contributed to the scientific concept, revised the manuscript, contributed to data interpretation, and performed the statistical analysis. YS, SK, AA-S, SM, DO and JS contributed to obtaining clinical data, and organized and implemented sample management. PK, RY and CM analyzed data and critically revised the manuscript. All authors contributed to the article and approved the submitted version.
