# Supplementary material for: Are IL-1 family cytokines important in management of sickle cell disease in Sub-Saharan Africa patients?
Source: Front Immunol. 2023 Mar 9;14:954054. doi: 10.3389/fimmu.2023.954054 (PMC10034065; doi:10.3389/fimmu.2023.954054)
Supplement: Supplementary file 1 [file DataSheet_1.docx]

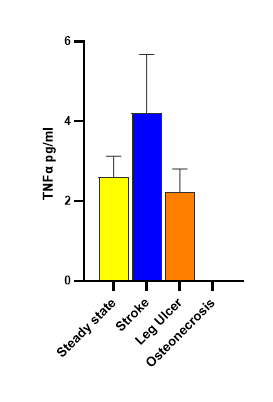


**Supplementary Figure S1. Levels of other cytokines according to the complications in SCD patients**

*Cytokine levels were assessed in steady state SCD patients (34 patients; yellow columns), in crisis patients with stroke (17 patients; blue columns), in leg ulcers (10 patients; orange columns) and in osteonecrosis (4 patients; grey columns). Data are presented as mean pg/ml ± SD. A statistically significant difference was found for TNFα (stroke vs. steady state). * p < 0.05*
