# Supplementary material for: Are IL-1 family cytokines important in management of sickle cell disease in Sub-Saharan Africa patients?
Source: Front Immunol. 2023 Mar 9;14:954054. doi: 10.3389/fimmu.2023.954054 (PMC10034065; doi:10.3389/fimmu.2023.954054)
Supplement: Supplementary file 2 [file DataSheet_2.docx]

**Supplementary Figure S2:** **Levels of other cytokines according to the type of crisis in SCD patients**

*The levels of IL-6 (upper left), IL-8 (upper right), IL10 (lower left) and TNFa (lower right) in SCD patients were compared between steady state (34 patients; yellow columns), vaso-occlusive crisis (VOC; 32 patients; green columns) and hemolytic anemia (HA; 24 patients; brown columns).*

*Data are presented as median values and interquartile range in pg/ml. Statistical analysis was performed using Kruskal-Wallis. * p<0.05*
