# Supplementary material for: Are IL-1 family cytokines important in management of sickle cell disease in Sub-Saharan Africa patients?
Source: Front Immunol. 2023 Mar 9;14:954054. doi: 10.3389/fimmu.2023.954054 (PMC10034065; doi:10.3389/fimmu.2023.954054)
Supplement: Supplementary file 3 [file Table_1.docx]

**Supplementary table S1 : Levels of other cytokines in SCD patients**

| **Cytokines**  *(pg/ml)* | **Steady state**  *mean ± SD (min-max)* | **Crisis**  *mean ± SD (min-max)* | ***p value*** |
| --- | --- | --- | --- |
| **IFN-α2** | 53.79 ± 237.02 (0-1262.40) | 10.18 ± 5.67 (0-19.93) | 0.4 |
| **IFN-γ** | 7.27 ± 11.30 (0-44.39) | 6.64 ± 8.20 (0-29.79) | 0.8 |
| **MCP-1** | 239.00 ± 260.74 (27.17-1015.13) | 281.37 ± 670.26 (11.34-3530.89) | 0.7 |
| **IL-12p70** | 1.30 ± 2.14 (0-7.82) | 4.23 ± 9.14 (0-47.90) | 0.1 |
| **IL-17A** | 7.80 ± 7.48 (0-38.93) | 10.5 ± 11.84 (0-37.33) | 0.3 |
| **IL-23** | 11.26 ± 14.14 (0-65.19) | 24.70 ± 68.97 (0-372.48) | 0.3 |
